# XEC severe acute respiratory syndrome coronavirus 2 subvariant in Africa: A rising health alarm

**DOI:** 10.4102/jphia.v16i1.1235

**Published:** 2025-02-19

**Authors:** Abdulrakib Abdulrahim, Bashar H. Gulumbe, Michael A. Oluwasola, Mohammed B. Danlami, Yusuff A. Adebisi

**Affiliations:** 1 Department of Microbiology, Faculty of Science, Federal University Birnin Kebbi, Birnin Kebbi, Nigeria; 2 Department of Medical Microbiology, Faculty of Medicine and Health Sciences, Universiti Putra Malaysia, Serdang, Malaysia; 3 Department of Cell and Molecular Biology, Faculty of Biotechnology and Bimolecular Science, Universiti Putra Malaysia, Serdang, Malaysia; 4 College of Social Sciences, University of Glasgow, Glasgow, United Kingdom

Dear Editor,

The coronavirus disease 2019 (COVID-19), caused by severe acute respiratory syndrome coronavirus 2 (SARS-CoV-2), remains one of the most devastating public health crises in recent history, affecting millions worldwide. As of October 2024, the pandemic has resulted in over 774 million confirmed cases and more than 7 million confirmed deaths globally since its emergence in December 2019.^1^ SARS-CoV-2 variants by the World Health Organization (WHO): Alpha, Beta, Gamma, Delta and Omicron. These variants were first identified in the United Kingdom, South Africa, Brazil, India and Botswana, respectively.^1,2^ Over time, these variants have continued to evolve, producing numerous lineages and subvariants.^2^ Among these, Omicron subvariants, including BA.2.86, JN.1, KP.2, KP.3 and XEC, remain prevalent worldwide.^3^

Recently, a new subvariant, XEC, has emerged and continues to spread, with one case reported in Africa, amid ongoing public health challenges such as mpox, which was declared for the first time as a Public Health Emergency of Continental Security by the Africa Centers for Disease Control and Prevention (Africa CDC) in August 2024.^1,3,4^ This letter emphasises the urgent need to implement public health measures to curb the spread of the XEC subvariant in Africa (Table 1).

**TABLE 1:** Urgent measures to mitigate the spread of the SARS-CoV-2 XEC subvariant in Africa.

S/N	Key immediate interventions	Description
1.	Community engagement and education	Educate communities on the importance of early detection and prevention, addressing this new subvariant and the issue of misinformation.
2.	Enhanced border screening	Strengthen screening at airports and land borders for travellers from regions with reported XEC cases.
3.	Accelerated vaccination efforts and other preventive approaches	Boost COVID-19 vaccination campaigns and ensure equitable distribution, particularly in rural and underserved areas. Additionally, handwashing and face masks should be promoted in crowded places.
4.	Rapid response plans	Develop and deploy plans to address potential XEC subvariant outbreaks promptly.
5.	Strengthened surveillance networks	Improve networks to track transmission patterns and monitor potential outbreaks effectively.
6.	Collaboration with global health authorities	Enhance cooperation between African health authorities, WHO and Africa CDC for data sharing and resource allocation.

S/N, serial number; WHO, World Health Organization; CDC, Centers for Disease Control and Prevention; COVID-19, coronavirus disease 2019; SARS-CoV-2, severe acute respiratory syndrome coronavirus 2.

The XEC subvariant is a recombinant virus derived from merging two known Omicron lineages: JN.1:KP.3.3 and KS.1.1.^3,5^ This subvariant was first identified in Germany in May 2024 and is classified as a Variant Under Monitoring by the WHO because of its distinctive characteristics.^2,3^ Clinical manifestations of XEC are similar to those of other variants and include coughing, nasal congestion or a runny nose, diarrhoea, fever or chills, difficulty breathing and loss of taste or smell.^3,5^ The primary threat posed by XEC is its high transmissibility compared to other variants.^5^

A study by Kaku et al. found that the XEC subvariant has a higher effective reproduction number than other variants, suggesting it may become dominant globally shortly.^6^ As of late November, this new subvariant accounted for approximately 38% of COVID-19 infections in the United States, affecting over 50% of the states and ranking as the second most reported variant.^5,7^ It has spread to more than 40 countries across Asia, Africa, Europe and North America.^2,3^ Africa reported its first case of the XEC subvariant in early November 2024 in Botswana, involving a hospitalised European tourist. However, limited testing and sequencing capacity in Africa compared to the pandemic period hampers the ability to track and assess the subvariant’s spread in real time, which could undermine preparedness and response efforts.^3^ According to the CDC, the updated 2024–2025 COVID-19 vaccines, such as Pfizer and Moderna messenger ribonucleic acid (mRNA), may protect against currently circulating SARS-CoV-2 variants, including XEC.^7^ Additionally, JN.1 booster vaccination has shown effectiveness against the XEC lineage although its neutralisation efficacy is lower than the JN.1 lineage.^8^

## Conclusion

As a highly transmissible recombinant variant, XEC necessitates urgent, coordinated action to strengthen surveillance, vaccination coverage and public engagement. Limited diagnostic capacities must be addressed to prevent silent transmission. As emphasised by the Africa CDC, investments in testing and sequencing infrastructure during the COVID-19 pandemic must be sustained and expanded to monitor and respond to emerging threats. Support for training local experts, genomic surveillance and healthcare infrastructure is essential to bolster Africa’s readiness for future outbreaks. Maintaining and enhancing these capacities will contribute to global health security by curbing the emergence and spread of SARS-CoV-2 subvariants and other public health emergencies. Long-term funding and collaboration are critical to achieving these goals. No one is truly safe until everyone is safe.
